# (3a*S*,4*S*,6*S*,7a*R*)-Hexahydro-3a,5,5-trimethyl-2-phenyl-4,6-methano-1,3,2-benzodioxaborole

**DOI:** 10.1107/S1600536812041712

**Published:** 2012-10-13

**Authors:** Tore Lejon, Olga V. Gozhina, Victor N. Khrustalev

**Affiliations:** a Department of Chemistry, Faculty of Science and Technology, University of Tromsø, N-9037 Tromsø, Norway; b X-ray Structural Centre, A. N. Nesmeyanov Institute of Organoelement Compounds, Russian Academy of Sciences, 28 Vavilov Street B-334, Moscow 119991, Russian Federation

## Abstract

The mol­ecule of the title compound, C_16_H_21_BO_2_, comprises a chiral fused tricyclic system containing five-membered (1,3,2-dioxaborolane), six-membered (cyclo­hexa­ne) and four-membered (cyclo­butane) rings. The 1,3,2-dioxaborolane ring is almost planar (r.m.s. deviation = 0.035 Å), and the *syn* H and Me substituents at this ring are in an eclipsed conformation. The cyclo­hexane and cyclo­butane rings adopt sofa and butterfly conformations, respectively. The B atom has a trigonal–planar configuration (sum of the bond angles = 360.0°). The phenyl ring is practically coplanar with the 1,3,2-dioxaborolane ring [dihedral angle between the ring planes = 1.96 (8)°]. The absolute structure was determined from the known configuration of (+)-pinanediol which was used in the synthesis. In the crystal, weak C—H⋯π(Ph) inter­actions occur.

## Related literature

For the Matteson homologation reaction, see: Matteson *et al.* (1983 ▶); Matteson (1989 ▶). For 2-substituted (+)-pinanediolboronates, see: Carmès *et al.* (2000) ▶; Caselli *et al.* (2003 ▶); Morandi *et al.* (2003 ▶, 2005 ▶).
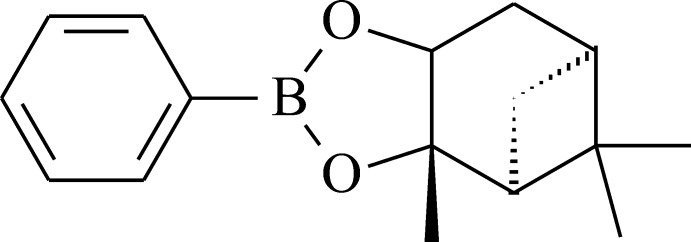


## Experimental

### 

#### Crystal data

C_16_H_21_BO_2_
*M* *_r_* = 256.14
Orthorhombic, 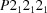

*a* = 8.4974 (3) Å
*b* = 11.8566 (4) Å
*c* = 13.9580 (4) Å
*V* = 1406.27 (8) Å^3^
*Z* = 4
Mo *K*α radiation
μ = 0.08 mm^−1^
*T* = 100 K
0.25 × 0.22 × 0.18 mm

#### Data collection

Bruker APEXII CCD diffractometer
Absorption correction: multi-scan (*SADABS*; Sheldrick, 2003 ▶) *T*_min_ = 0.981, *T*_max_ = 0.986
21778 measured reflections
2905 independent reflections
2717 reflections with *I* > 2σ(*I*)
*R*_int_ = 0.037

#### Refinement

*R*[*F* ^2^ > 2σ(*F* ^2^)] = 0.034
*wR*(*F* ^2^) = 0.092
*S* = 1.05
2905 reflections
176 parameters
H-atom parameters constrained
Δρ_max_ = 0.30 e Å^−3^
Δρ_min_ = −0.20 e Å^−3^

### 

Data collection: *APEX2* (Bruker, 2005 ▶); cell refinement: *SAINT-Plus* (Bruker, 2001 ▶); data reduction: *SAINT-Plus*; program(s) used to solve structure: *SHELXTL* (Sheldrick, 2008 ▶); program(s) used to refine structure: *SHELXTL*; molecular graphics: *SHELXTL*; software used to prepare material for publication: *SHELXTL*.

## Supplementary Material

Crystal structure: contains datablock(s) global, I. DOI: 10.1107/S1600536812041712/fb2267sup1.cif

Structure factors: contains datablock(s) I. DOI: 10.1107/S1600536812041712/fb2267Isup2.hkl

Additional supplementary materials:  crystallographic information; 3D view; checkCIF report

## Figures and Tables

**Table 1:** Hydrogen-bond geometry (Å, °) *Cg* is the centroid of the C12–C17 phenyl ring

*D*—H⋯*A*	*D*—H	H⋯*A*	*D*⋯*A*	*D*—H⋯*A*
C8—H8*A*⋯*Cg* ^i^	0.99	2.94	3.7532 (13)	140
C11—H11*A*⋯*Cg* ^ii^	0.98	2.97	3.9481 (13)	174

Symmetry codes: (i) 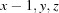; (ii) 
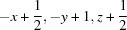
.

**Fig. 1.**
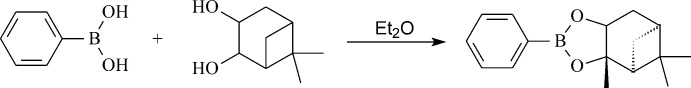
Reaction of phenylboronic acid with (+)-pinanediol.

**Fig. 2.**
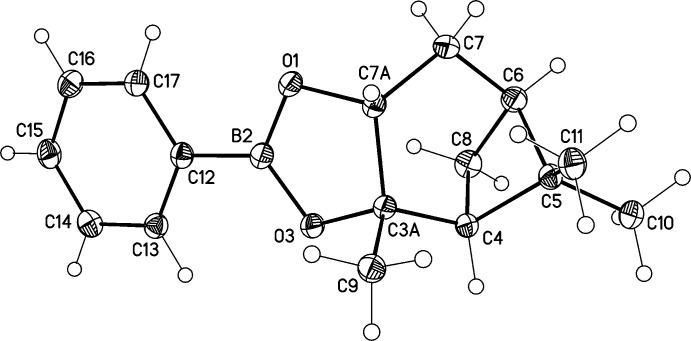
The title molecule with the atom numbering scheme. The displacement ellipsoids are shown at the 50% probability level. H atoms are presented as small spheres of arbitrary radius.

## supplementary crystallographic information

### Comment

2-Substituted (+)-pinanediolboronates are well known compounds (Carmès *et al.*, 2000; Caselli *et al.*, 2003; Morandi *et al.*, 2003; Morandi *et al.*, 2005). In the present work, the (+)-pinanediol phenylboronate (CAS Registry Number: 76110-78-6) has been synthesized by the reaction of phenylboronic acid with (+)-pinanediol in diethyl ether (Fig. 1) as a starting material for the Matteson homologation reaction (Matteson *et al.*, 1983; Matteson, 1989).

The molecule of the title compound, C_16_H_21_BO_2_, comprises a chiral fused tricyclic system containing five-membered (1,3,2-dioxaborolane), six-membered (cyclohexane) and four-membered (cyclobutane) rings (Fig. 2). The 1,3,2-dioxaborolane ring is almost planar (s.u. = 0.035 Å), and *syn* hydrogen and methyl substituents at this ring are in an eclipsed conformation. The cyclohexane (C8/C4/C3a/C7a/C7/C6 and C5/C4/C3a/C7a/C7/C6) and cyclobutane rings adopt *sofa* and *butterfly* conformations, respectively. The boron atom has a trigonal-planar configuration (sum of the bond angles is 360.0 (3)°). The phenyl ring is practically coplanar to the 1,3,2-dioxaborolane ring (the interplanar angle between the ring planes is 1.96 (8)°).

The absolute structure was determined from the known configuration of (+)-pinanediol that has been used in the synthesis. The title molecule possesses four asymmetric centres at C3a, C4, C6 and C7a carbon atoms. The configurations at these asymmetric centres are *S*, *S*, *S*, *R*, respectively.

The crystal packing of the title molecules is stabilized by the weak intermolecular C8—H8A···π(Ph) [the H···*Cg*^i^ distance is 2.94 Å; *Cg* means the centroid of the phenyl ring composed of C12/C13–C17] and C11—H11A···π(Ph) [the H···*Cg*^ii^ distance is 2.97 Å] interactions. Symmetry codes: (i) -1 + *x*, *y*, *z*; (ii) 1/2 - *x*, 1 - *y*, 1/2 + *z*.

### Experimental

(+)-Pinanediol (10 g, 0.059 mol) was added to a solution of phenylboronic acid (7.16 g, 0.059 mol) in ether (80 ml). The reaction mixture was stirred for 4 h at room temperature. Then the solvent was evaporated under reduced pressure to give the title compound as colourless prismatic crystals. Yield is 15 g (99%). ^1^H NMR (400 MHz, CDCl_3_): δ = 8.25 (dd, *J* = 8.0, 1.4 Hz, 1H), 7.83–7.80 (m, 1H), 7.63–7.35 (m, 3H), 4.46 (dd, *J* = 8.8, 1.9 Hz, 1H), 2.41 (ddd, *J* = 8.6, 8.0, 5.6 Hz,1*H*), 2.23 (ddd, *J* = 10.8, 6.1, 2.3 Hz, 1H), 2.15 (d, *J* = 5.9 Hz, 1H), 1.96 (ddd, *J* = 12.2, 5.5, 3.0 Hz, 2H), 1.49 (s, 3H), 1.32 (s, 3H), 1.23 (d, *J* = 10.8 Hz, 1H), 0.90 (s, 3H). ^13^C NMR (101 MHz, CDCl_3_): δ = 135.62, 134.76, 132.68, 131.15, 127.97, 127.72, 86.22, 78.24, 51.42, 39.54, 38.19, 35.57, 28.71, 27.10, 26.49, 24.04.

### Refinement

All the H atoms were discernible in the difference electron density map. Nevertheless, all the H atoms were fully constrained. The values of the used constraints were following: C_aryl_—H = 0.95, C_methyl_—H = 0.98, C_methylene_—H = 0.99, C_methine_—H = 1.00 Å; *U*_iso_(H) = 1.2*U*_eq_(C_aryl/methylene/methine_); *U*_iso_(H) = 1.5*U*_eq_(C_methyl_). After the refinement converged the extremal residual density peaks equalled to -0.197 and 0.302 e A^-3^. All the highest positive peaks in the range of 0.170–0.302 e A^-3^) were situated between the covalently bonded atoms. 2225 Friedel pairs were merged in the refinement process. The absolute structure was determined from the known configuration of (+)-pinanediol that had been used in the synthesis.

### Crystal data

**Table:**

C_16_H_21_BO_2_	*F*(000) = 552
*M**_r_* = 256.14	*D*_x_ = 1.210 Mg m^−^^3^
Orthorhombic, *P*2_1_2_1_2_1_	Mo *K*α radiation, λ = 0.71073 Å
Hall symbol: P 2ac 2ab	Cell parameters from 7519 reflections
*a* = 8.4974 (3) Å	θ = 2.3–32.2°
*b* = 11.8566 (4) Å	µ = 0.08 mm^−^^1^
*c* = 13.9580 (4) Å	*T* = 100 K
*V* = 1406.27 (8) Å^3^	Prism, colourless
*Z* = 4	0.25 × 0.22 × 0.18 mm

### Data collection

**Table:**

Bruker APEXII CCD diffractometer	2905 independent reflections
Radiation source: fine-focus sealed tube	2717 reflections with *I* > 2σ(*I*)
Graphite monochromator	*R*_int_ = 0.037
φ and ω scans	θ_max_ = 32.7°, θ_min_ = 2.3°
Absorption correction: multi-scan (*SADABS*; Sheldrick, 2003)	*h* = −12→12
*T*_min_ = 0.981, *T*_max_ = 0.986	*k* = −17→18
21778 measured reflections	*l* = −21→21

### Refinement

**Table:**

Refinement on *F*^2^	Secondary atom site location: difference Fourier map
Least-squares matrix: full	Hydrogen site location: difference Fourier map
*R*[*F*^2^ > 2σ(*F*^2^)] = 0.034	H-atom parameters constrained
*wR*(*F*^2^) = 0.092	*w* = 1/[σ^2^(*F*_o_^2^) + (0.0609*P*)^2^ + 0.0967*P*] where *P* = (*F*_o_^2^ + 2*F*_c_^2^)/3
*S* = 1.05	(Δ/σ)_max_ < 0.001
2905 reflections	Δρ_max_ = 0.30 e Å^−^^3^
176 parameters	Δρ_min_ = −0.20 e Å^−^^3^
0 restraints	Extinction correction: *SHELXL97* (Sheldrick, 2008), Fc^*^=kFc[1+0.001xFc^2^λ^3^/sin(2θ)]^-1/4^
Primary atom site location: structure-invariant direct methods	Extinction coefficient: 0.015 (3)

### Special details

**Table:**

Geometry. All e.s.d.'s (except the e.s.d. in the dihedral angle between two l.s. planes) are estimated using the full covariance matrix. The cell e.s.d.'s are taken into account individually in the estimation of e.s.d.'s in distances, angles and torsion angles; correlations between e.s.d.'s in cell parameters are only used when they are defined by crystal symmetry. An approximate (isotropic) treatment of cell e.s.d.'s is used for estimating e.s.d.'s involving l.s. planes.
Refinement. Refinement of *F*^2^ against ALL reflections. The weighted *R*-factor *wR* and goodness of fit *S* are based on *F*^2^, conventional *R*-factors *R* are based on *F*, with *F* set to zero for negative *F*^2^. The threshold expression of *F*^2^ > σ(*F*^2^) is used only for calculating *R*-factors(gt) *etc*. and is not relevant to the choice of reflections for refinement. *R*-factors based on *F*^2^ are statistically about twice as large as those based on *F*, and *R*- factors based on ALL data will be even larger.

### Fractional atomic coordinates and isotropic or equivalent isotropic displacement parameters (Å^2^)

**Table:**

	*x*	*y*	*z*	*U*_iso_*/*U*_eq_	
O1	0.47304 (10)	0.46977 (7)	0.16126 (6)	0.01697 (16)	
B2	0.51357 (15)	0.50773 (11)	0.07183 (9)	0.0155 (2)	
O3	0.43471 (10)	0.60223 (7)	0.04344 (6)	0.01612 (16)	
C3A	0.33535 (12)	0.64035 (9)	0.12251 (7)	0.01379 (18)	
C4	0.16707 (12)	0.65105 (9)	0.08556 (7)	0.01398 (18)	
H4	0.1503	0.7117	0.0368	0.017*	
C5	0.04178 (12)	0.64743 (9)	0.16843 (8)	0.01482 (18)	
C6	0.05741 (14)	0.51655 (9)	0.16181 (8)	0.0170 (2)	
H6	−0.0424	0.4734	0.1719	0.020*	
C7	0.19909 (14)	0.47449 (10)	0.21897 (8)	0.0181 (2)	
H7A	0.2184	0.3944	0.2029	0.022*	
H7B	0.1743	0.4788	0.2882	0.022*	
C7A	0.35083 (13)	0.54313 (9)	0.19900 (7)	0.01454 (19)	
H7C	0.3886	0.5765	0.2606	0.017*	
C8	0.10773 (14)	0.53195 (10)	0.05572 (8)	0.0182 (2)	
H8A	0.0190	0.5329	0.0097	0.022*	
H8B	0.1920	0.4800	0.0346	0.022*	
C9	0.40689 (14)	0.75145 (9)	0.15593 (8)	0.0180 (2)	
H9A	0.4052	0.8059	0.1031	0.027*	
H9B	0.5158	0.7388	0.1763	0.027*	
H9C	0.3457	0.7811	0.2098	0.027*	
C10	−0.11904 (13)	0.68839 (11)	0.13230 (9)	0.0195 (2)	
H10A	−0.1992	0.6736	0.1812	0.029*	
H10B	−0.1464	0.6482	0.0733	0.029*	
H10C	−0.1142	0.7696	0.1194	0.029*	
C11	0.07336 (14)	0.70381 (10)	0.26519 (8)	0.0189 (2)	
H11A	−0.0121	0.6856	0.3097	0.028*	
H11B	0.0791	0.7857	0.2566	0.028*	
H11C	0.1733	0.6762	0.2912	0.028*	
C12	0.63777 (13)	0.44754 (9)	0.00804 (8)	0.01522 (19)	
C13	0.68207 (13)	0.49218 (10)	−0.08098 (8)	0.0167 (2)	
H13	0.6353	0.5602	−0.1029	0.020*	
C14	0.79409 (15)	0.43795 (10)	−0.13785 (8)	0.0191 (2)	
H14	0.8243	0.4695	−0.1976	0.023*	
C15	0.86127 (14)	0.33749 (10)	−0.10656 (9)	0.0202 (2)	
H15	0.9356	0.2995	−0.1459	0.024*	
C16	0.82009 (15)	0.29237 (10)	−0.01792 (9)	0.0219 (2)	
H16	0.8673	0.2243	0.0037	0.026*	
C17	0.70942 (14)	0.34746 (10)	0.03884 (8)	0.0186 (2)	
H17	0.6820	0.3167	0.0994	0.022*	

### Atomic displacement parameters (Å^2^)

**Table:**

	*U*^11^	*U*^22^	*U*^33^	*U*^12^	*U*^13^	*U*^23^
O1	0.0158 (4)	0.0176 (4)	0.0175 (3)	0.0048 (3)	0.0017 (3)	0.0028 (3)
B2	0.0141 (5)	0.0152 (5)	0.0171 (5)	0.0012 (4)	0.0001 (4)	−0.0003 (4)
O3	0.0159 (4)	0.0166 (3)	0.0159 (3)	0.0034 (3)	0.0031 (3)	0.0019 (3)
C3A	0.0135 (4)	0.0135 (4)	0.0144 (4)	0.0010 (4)	0.0011 (3)	0.0007 (3)
C4	0.0129 (4)	0.0138 (4)	0.0152 (4)	−0.0002 (3)	0.0001 (3)	0.0006 (3)
C5	0.0127 (4)	0.0146 (4)	0.0172 (4)	−0.0005 (4)	0.0003 (4)	−0.0001 (4)
C6	0.0153 (4)	0.0139 (4)	0.0217 (5)	−0.0018 (4)	−0.0006 (4)	0.0010 (4)
C7	0.0167 (5)	0.0157 (4)	0.0218 (5)	−0.0006 (4)	0.0016 (4)	0.0047 (4)
C7A	0.0140 (4)	0.0145 (4)	0.0151 (4)	0.0020 (4)	0.0010 (3)	0.0008 (4)
C8	0.0187 (5)	0.0169 (5)	0.0191 (5)	−0.0017 (4)	−0.0019 (4)	−0.0036 (4)
C9	0.0157 (5)	0.0151 (4)	0.0231 (5)	−0.0019 (4)	0.0004 (4)	−0.0004 (4)
C10	0.0143 (5)	0.0214 (5)	0.0228 (5)	0.0020 (4)	−0.0003 (4)	0.0026 (4)
C11	0.0182 (5)	0.0203 (5)	0.0182 (5)	0.0007 (4)	0.0022 (4)	−0.0024 (4)
C12	0.0138 (4)	0.0151 (4)	0.0167 (4)	−0.0001 (4)	−0.0001 (4)	−0.0014 (4)
C13	0.0158 (5)	0.0170 (5)	0.0173 (4)	0.0002 (4)	−0.0008 (4)	−0.0004 (4)
C14	0.0191 (5)	0.0213 (5)	0.0169 (5)	−0.0010 (4)	0.0014 (4)	−0.0021 (4)
C15	0.0179 (5)	0.0207 (5)	0.0220 (5)	0.0013 (4)	0.0040 (4)	−0.0043 (4)
C16	0.0205 (5)	0.0180 (5)	0.0272 (5)	0.0054 (4)	0.0036 (4)	0.0004 (4)
C17	0.0175 (5)	0.0180 (5)	0.0204 (5)	0.0031 (4)	0.0034 (4)	0.0015 (4)

### Geometric parameters (Å, º)

**Table:**

O1—B2	1.3709 (15)	C8—H8B	0.9900
O1—C7A	1.4535 (13)	C9—H9A	0.9800
B2—O3	1.3644 (15)	C9—H9B	0.9800
B2—C12	1.5544 (16)	C9—H9C	0.9800
O3—C3A	1.4612 (13)	C10—H10A	0.9800
C3A—C9	1.5239 (15)	C10—H10B	0.9800
C3A—C4	1.5254 (15)	C10—H10C	0.9800
C3A—C7A	1.5767 (15)	C11—H11A	0.9800
C4—C8	1.5563 (16)	C11—H11B	0.9800
C4—C5	1.5727 (15)	C11—H11C	0.9800
C4—H4	1.0000	C12—C17	1.4012 (15)
C5—C11	1.5307 (15)	C12—C13	1.4020 (15)
C5—C10	1.5355 (15)	C13—C14	1.3963 (16)
C5—C6	1.5602 (15)	C13—H13	0.9500
C6—C7	1.5280 (16)	C14—C15	1.3911 (17)
C6—C8	1.5520 (17)	C14—H14	0.9500
C6—H6	1.0000	C15—C16	1.3927 (17)
C7—C7A	1.5500 (16)	C15—H15	0.9500
C7—H7A	0.9900	C16—C17	1.3924 (16)
C7—H7B	0.9900	C16—H16	0.9500
C7A—H7C	1.0000	C17—H17	0.9500
C8—H8A	0.9900		
			
B2—O1—C7A	108.24 (9)	C6—C8—H8A	114.2
O3—B2—O1	114.25 (10)	C4—C8—H8A	114.2
O3—B2—C12	122.96 (10)	C6—C8—H8B	114.2
O1—B2—C12	122.78 (10)	C4—C8—H8B	114.2
B2—O3—C3A	108.56 (8)	H8A—C8—H8B	111.4
O3—C3A—C9	105.55 (8)	C3A—C9—H9A	109.5
O3—C3A—C4	108.18 (8)	C3A—C9—H9B	109.5
C9—C3A—C4	113.93 (9)	H9A—C9—H9B	109.5
O3—C3A—C7A	103.72 (8)	C3A—C9—H9C	109.5
C9—C3A—C7A	113.04 (9)	H9A—C9—H9C	109.5
C4—C3A—C7A	111.59 (9)	H9B—C9—H9C	109.5
C3A—C4—C8	108.59 (9)	C5—C10—H10A	109.5
C3A—C4—C5	112.56 (8)	C5—C10—H10B	109.5
C8—C4—C5	87.29 (8)	H10A—C10—H10B	109.5
C3A—C4—H4	115.1	C5—C10—H10C	109.5
C8—C4—H4	115.1	H10A—C10—H10C	109.5
C5—C4—H4	115.1	H10B—C10—H10C	109.5
C11—C5—C10	107.92 (9)	C5—C11—H11A	109.5
C11—C5—C6	118.15 (9)	C5—C11—H11B	109.5
C10—C5—C6	111.77 (9)	H11A—C11—H11B	109.5
C11—C5—C4	121.22 (9)	C5—C11—H11C	109.5
C10—C5—C4	110.62 (9)	H11A—C11—H11C	109.5
C6—C5—C4	85.75 (8)	H11B—C11—H11C	109.5
C7—C6—C8	108.63 (10)	C17—C12—C13	118.35 (10)
C7—C6—C5	111.14 (9)	C17—C12—B2	120.53 (10)
C8—C6—C5	87.88 (8)	C13—C12—B2	121.11 (10)
C7—C6—H6	115.3	C14—C13—C12	120.87 (11)
C8—C6—H6	115.3	C14—C13—H13	119.6
C5—C6—H6	115.3	C12—C13—H13	119.6
C6—C7—C7A	112.97 (9)	C15—C14—C13	119.71 (11)
C6—C7—H7A	109.0	C15—C14—H14	120.1
C7A—C7—H7A	109.0	C13—C14—H14	120.1
C6—C7—H7B	109.0	C14—C15—C16	120.31 (11)
C7A—C7—H7B	109.0	C14—C15—H15	119.8
H7A—C7—H7B	107.8	C16—C15—H15	119.8
O1—C7A—C7	110.20 (9)	C17—C16—C15	119.67 (11)
O1—C7A—C3A	104.58 (8)	C17—C16—H16	120.2
C7—C7A—C3A	115.87 (9)	C15—C16—H16	120.2
O1—C7A—H7C	108.7	C16—C17—C12	121.07 (11)
C7—C7A—H7C	108.7	C16—C17—H17	119.5
C3A—C7A—H7C	108.7	C12—C17—H17	119.5
C6—C8—C4	86.60 (8)		
			
C7A—O1—B2—O3	1.42 (13)	B2—O1—C7A—C7	119.37 (10)
C7A—O1—B2—C12	−178.09 (10)	B2—O1—C7A—C3A	−5.80 (11)
O1—B2—O3—C3A	4.09 (13)	C6—C7—C7A—O1	−120.17 (10)
C12—B2—O3—C3A	−176.40 (10)	C6—C7—C7A—C3A	−1.72 (14)
B2—O3—C3A—C9	111.83 (10)	O3—C3A—C7A—O1	7.86 (10)
B2—O3—C3A—C4	−125.86 (10)	C9—C3A—C7A—O1	−105.95 (10)
B2—O3—C3A—C7A	−7.25 (11)	C4—C3A—C7A—O1	124.09 (9)
O3—C3A—C4—C8	63.98 (10)	O3—C3A—C7A—C7	−113.64 (10)
C9—C3A—C4—C8	−178.99 (9)	C9—C3A—C7A—C7	132.54 (10)
C7A—C3A—C4—C8	−49.50 (11)	C4—C3A—C7A—C7	2.59 (13)
O3—C3A—C4—C5	158.89 (8)	C7—C6—C8—C4	−85.15 (9)
C9—C3A—C4—C5	−84.08 (11)	C5—C6—C8—C4	26.47 (8)
C7A—C3A—C4—C5	45.42 (12)	C3A—C4—C8—C6	86.66 (9)
C3A—C4—C5—C11	37.66 (14)	C5—C4—C8—C6	−26.25 (8)
C8—C4—C5—C11	146.68 (10)	O3—B2—C12—C17	−176.74 (11)
C3A—C4—C5—C10	165.43 (9)	O1—B2—C12—C17	2.72 (17)
C8—C4—C5—C10	−85.55 (10)	O3—B2—C12—C13	3.88 (17)
C3A—C4—C5—C6	−82.88 (10)	O1—B2—C12—C13	−176.66 (11)
C8—C4—C5—C6	26.13 (8)	C17—C12—C13—C14	0.39 (17)
C11—C5—C6—C7	−40.37 (13)	B2—C12—C13—C14	179.78 (11)
C10—C5—C6—C7	−166.49 (9)	C12—C13—C14—C15	0.88 (17)
C4—C5—C6—C7	82.98 (10)	C13—C14—C15—C16	−1.55 (18)
C11—C5—C6—C8	−149.55 (10)	C14—C15—C16—C17	0.94 (19)
C10—C5—C6—C8	84.33 (10)	C15—C16—C17—C12	0.36 (19)
C4—C5—C6—C8	−26.20 (8)	C13—C12—C17—C16	−1.01 (17)
C8—C6—C7—C7A	47.70 (12)	B2—C12—C17—C16	179.59 (11)
C5—C6—C7—C7A	−47.38 (13)		

### Hydrogen-bond geometry (Å, º)

*Cg* is the centroid of the C12–C17 phenyl ring

**Table:**

*D*—H···*A*	*D*—H	H···*A*	*D*···*A*	*D*—H···*A*
C8—H8*A*···*Cg*^i^	0.99	2.94	3.7532 (13)	140
C11—H11*A*···*Cg*^ii^	0.98	2.97	3.9481 (13)	174

Symmetry codes: (i) *x*−1, *y*, *z*; (ii) −*x*+1/2, −*y*+1, *z*+1/2.
